# Electronic nose for odor monitoring at a landfill fenceline: Training and validation of a model for real-time odor concentration measurement

**DOI:** 10.1016/j.heliyon.2024.e31103

**Published:** 2024-05-10

**Authors:** Beatrice Julia Lotesoriere, Carmen Bax, Laura Capelli

**Affiliations:** Politecnico di Milano, Department of Chemistry, Materials and Chemical Engineering “Giulio Natta”, 20133, Milano, Italy

**Keywords:** IOMS, Odor concentration, Environmental monitoring, Performance verification, Uncertainty, Quantification

## Abstract

In recent years, electronic noses, or more generally Instrumental Odor Monitoring Systems (IOMS), have aroused increasing interest in the field of environmental monitoring.

One of the most interesting applications of these instruments is the real-time estimation of the odor concentration at plant fencelines to continuously monitor odor emissions and identify anomalous conditions. In this type of application, it is possible to setting a “warning” threshold, enabling the continuous check of proper functioning of the plant and sudden intervention in case of malfunctions, preventing, at the same time, the risk of odor events at the receptors. For this purpose, it is necessary to provide a continuous, fast and reliable measurement of the odor concentration, which is nowadays one of the main challenges of this technology.

In this context, this work proposes the development of a quantification model for quantifying odors detected at the fenceline of a landfill characterized by very different odor fingerprints. A double-step quantification model, firstly identifying the different odor classes to which the ambient air monitored at the fenceline by the IOMS belong to, and then developing different specific PLS regression models for each of the odor classes identified, was developed. The results of the proposed quantification model were compared to the ones obtained developing a “global” quantification model, which implements the regression on the globality of the training set, without differentiating between the odor classes. Then, they were further evaluated by comparison with the odor events detected at the sensitive receptor by another electronic nose. Moreover, the combined evaluation of the odor events at the plant fenceline and the receptor, respectively, together with the meteorological data highlighted the need of identifying variable warning thresholds for the odor concentrations at the fenceline according to effectively account for meteorological conditions and produce an output that is more correlated with the probability that an odor is perceived outside of the plant.

## Highlights

• Novel approach for real-time monitoring of odor concentration at fenceline.
• Proposed approach significantly improves the accuracy of the estimations by e-nose.
• Effective testing protocol for assessing e-nose performance in the field.
• Definition of alarm threshold for odor concentration for plant management.
• Alarm thresholds can prevent occurrence of odor events nearby the plant.

## 1 Introduction

Electronic noses (e-noses), currently referred to as Instrumental Odor Monitoring Systems (IOMS) have gained great popularity for environmental monitoring [[1], [2], [3], [4], [5], [6], [7]] thanks to reduced cost, portability, and capability to provide continuous data, enabling direct assessment of odor impact, even in case of complex, discontinuous, variable and extensive odor sources [[8], [9], [10], [11], [12], [13]]. Compared with most traditional odor measurement methods [7,14,15], e-noses represent a promising perspective. They can be installed directly where the odor presence is lamented for continuously acquiring data, which, depending on application and e-noses design [[16], [17], [18], [19], [20], [21]], can be either real-time or post-processed. Behaving as a black box, e-noses can be trained without requiring detailed information about chemical composition of the mixture under examination.

A very recent IOMS application for environmental monitoring concerns their installation at plant fenceline for a real-time estimation of the odor concentration, which is becoming more and more frequent in the environmental permit of waste treatment plants (WTP). The use of e-noses suitable for quantification purposes are far from being state-of-the art yet, because of the lack of standardization in experimental procedures for their use in the field [[22], [23], [24], [25]].

Many approaches for building IOMS quantification models [[26], [27], [28], [29]] have been proposed in the literature. Their common aim is to define a regressor, based on various algorithms, e.g., Artificial Neural Networks (ANN), Support Vector Regression (SVR), or Partial Least Squares regression (PLS), as a continuous mapping between sensors responses and odor concentration without investigating the potential interference of the odor type on the quantification performance [[30], [31], [32], [33], [34], [35], [36]].

In this context, this paper proposes a novel approach for odor quantification. It involves a double-step model, which first operates the classification of unknown samples, and then estimates the concentration of recognized odor based on the specific regression developed for that class.

When the chemical composition of the odors differs significantly depending on the source, as it is typically the case for WTP, samples having the same odor concentration may interact very differently with the sensor array, resulting in very diverse odor fingerprints. This in turn can affect the IOMS quantification, giving that the computation of specific regressions for each odor class may result in a more effective approach. To investigate this aspect, this paper compares the odor quantification performance achieved by an e-nose installed at the fenceline of a landfill based on a double-step quantification model with a global quantification model, which directly applies a regression algorithm on the whole dataset.

This paper also describes the approach developed to validate e-noses performance directly “in situ”, representing a crucial aspect currently under discussion by technical standardization groups, such as the European CEN TC264/WG41 and the Italian UNI, which recently published the technical standard UNI 11761:2023 (Italy), and IEEE P252, to ensure the reliability of IOMS outputs [22]. Since the verification of classification performance has been extensively discussed in other works [[37], [38], [39], [40]], this paper focuses on the evaluation of the quantification performance.

Finally, as a possible future development, this study investigates the possibility to define a *warning* threshold for odor concentration at fenceline to monitor the regular plant functioning and identify those situations that may cause odor events at receptors. To do this, another e-nose was installed at a receptor, located 2 km from the source along the main wind direction, to investigate whether higher concentrations measured at plant fenceline would correspond to the detection of odors outside the plant.

## 2 Material and methods

### 2.1 E-noses

**Fig. 1:**
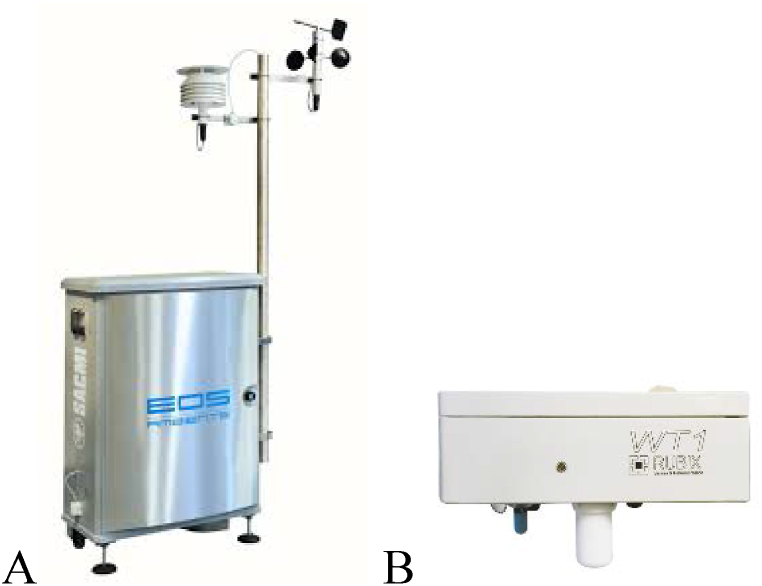
The two electronic noses used for the study: EOS 507F, commercialized by SACMI s.c. (A); WT1, commercialized by Ellona (B).

The hardware of two commercial e-noses were used (Fig. 1):

• EOS 507F (produced by SACMI) with 6 Metal Oxide Semiconductor (MOS) sensors, and an automatic system for humidity regulation allowing outdoor use even in the presence of variable weather conditions [41,42]. This instrument is characterized by high sensitivity towards very diluted odors, making it suitable for use at far distance from emissions sources. During operation, the instrument analyses ambient air at a suction rate of 25 mL/min and at a frequency of 1/60 Hz (Fig. 1A).
• WT1 (produced by Ellona) with 4 MOS sensors and 2 electrochemical sensors for H_2_S and NH_3_. The instrument comprises also four sensors for measuring temperature and relative humidity of the sensor chamber and of the external environment. The WT1 has a suction rate of approximately 1 L/min and provides responses at a frequency of 0.1 Hz (Fig. 1B).

The two instruments return the real-time responses of the sensors of the array (i.e., electrical resistance for MOS sensors and analytical concentrations in ppm for H_2_S and NH_3_ sensors), which were used as input of a data processing procedure, specifically developed for the application on RStudio (RStudio Team (2020). RStudio: Integrated Development for R. RStudio, PBC, Boston, MA URL http://www.rstudio.com/).

### 2.2 Case study description

The selected case study was developed at an Italian landfill for non-hazardous waste. The landfill consists of permanently and temporarily covered areas (i.e., covered with definitive coverage or waterproof polymeric sheets) and cultivation areas where waste arrival is still in progress, producing leachate and landfill gas, the latter being sent to an energy recovery plant.

**Fig. 2:**
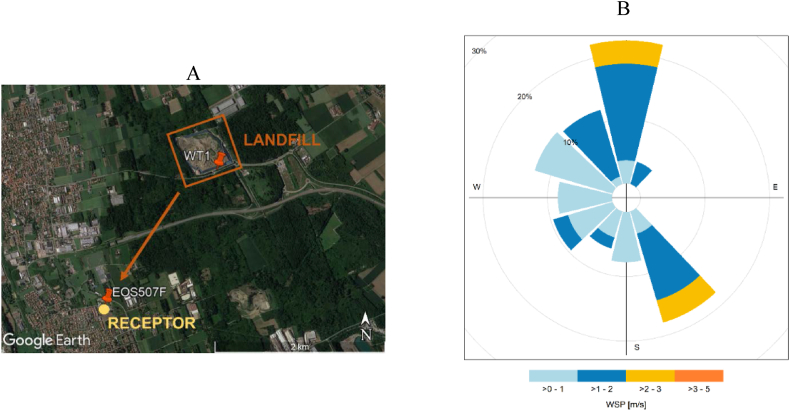
Google Earth map illustrating the landfill under investigation and monitoring sites for the two e-noses (i.e., WT1 and EOS 507F) (A); Wind rose relevant to the monitoring period, representing the wind provenance and relevant wind speed ranges (WSP) (B).

This paper presents the approach adopted to develop a system to continuously monitor odor emissions with two e-noses, used in a complementary way (Fig. 2A):

• The WT1 was installed at landfill fenceline to detect, classify, and quantify odors. This paper focuses mainly on odor quantification, further exploring the possibility to identify *warning* odor concentration levels at the fenceline, which might be associated with odor events at the closest receptors placed along the same direction.
• The EOS 507F was installed at a receptor located 2 km South of the landfill to continuously analyze the ambient air, detect the presence of odors, recognize whether they could be attributable to the landfill under investigation or not and investigate the correlation between the odor concentration at landfill fenceline with the occurrence of odor events at the receptor.

The choice of the monitoring sites was based on the results of previous odor characterization campaigns carried out at the plant and the history of citizens’ complaints reported to local authorities. Moreover, the analysis of the topography (predominantly flat area) and meteorological conditions of the area under study was considered (Fig. 2B). The site of the landfill is characterized by weak winds (speed < 2 m/s) with two prevalent directions: the main blowing from North to South, thus favoring the detection of landfill odors at monitored receptor, and another blowing from South-East to North-West.

### 2.3 Training

#### 2.3.1 Experimental protocol

The training consists in the creation of the training set (TS) comprising characteristic patterns of the odors that e-noses will be exposed to during monitoring. Thus, training will be different depending on the e-nose final use. Training for classification usually entails the analysis of samples belonging to the different odor classes under examination. For quantification, e-nose should be trained with samples having different odor concentrations to enable the construction of suitable regression models. In this work, the WT1 was trained for both odor classification and quantification. Conversely, the EOS 507F was trained only for classification because odor concentration measurement in ambient air at so far distance from the source would be useless [38].

The training involved the collection of samples representatives of main landfill odor sources, which were defined based on previous olfactometric campaigns carried out at the plant: fresh waste disposal, landfill gas emitted from landfill surface, and leachate collection tanks [[43], [44], [45]]. Samples were collected at emission sources (according to olfactometric sampling protocols [46]) on different days, characterized by different meteorological conditions (i.e. sunny and foggy days), to include in the TS the intrinsic variability of landfill emissions. After collection, they were analyzed by dynamic olfactometry to assess their odor concentration [47]. Based on their concentration, odor samples were diluted with ambient air and presented to the IOMS at different concentration levels to build the TS. As a general rule, the concentration range to be considered for training should be representative of concentration levels that e-noses in the field will be exposed to, which are obviously lower than concentrations at emissions due to atmospheric dilution [40]. 3 to 5 diluted samples were prepared for each odor sample collected at the emission sources and analyzed by the IOMS during the training phase. Dynamic olfactometry and IOMS analyses were carried out within 30 h following sampling, according to EN 13725:2022. In general, the time difference between the two analyses was of about 2–3 h. Odor samples were presented to the IOMS at increasing concentrations, to assess the Lower Detection Limit (LDL) towards the main landfill odors. The LDL, expressed in ou_E_/m^3^ and defined as the lowest concentration of a given substance/sample at which the e-nose response exceeds the condition of neutrality (LOD), provides information about e-nose sensitivity [40]. Combining the information about the instrument LDL with the characteristics of the monitoring sites (e.g., distance from odor sources) allowed to define odor concentration ranges to be considered for training.

During training, also non-odorous ambient air samples collected in the field, when no odor was perceivable by operators, were analyzed to define the LOD [38]. Thus, the TS comprises 24 odorless samples collected in ambient air at the WTP and 28 samples collected directly at WTP emission sources from which diluted samples were prepared, leading to include in the TS four classes: Air, Fresh Waste, Landfill Gas and Leachate.

#### 2.3.2 Data processing

First, training data were organized in a data frame combining features vectors extracted from raw sensors responses with samples information (i.e., label and odor concentration), as described in section 2.1 E-noses.

Then, data were processed by Principal Component Analysis (PCA) to explore dataset structure, identify and remove outliers (i.e., evaluated as the points lying more than 2 or 3 standard deviations from the mean of the data), and obtain a graphical visualization of the e-nose discrimination capability between the different classes [48].

For building classification models on training data, k-NN [49] and StepWise Linear regression (SW Linear) [50] algorithms were used for WT1 and EOS 507F, respectively. The SW Linear model is a method of regressing multiple variables while simultaneously removing those that are not important. Step-Wise regression essentially does multiple regression a number of times, each time removing the weakest correlated variable. At the end, only the variables that best explain the distribution remain [50]. The k-NN model estimates the class of unknown samples by a plurality vote of its neighbors, with the object being assigned to the most common class among its k nearest neighbors. Thus, the building of k-NN classifiers requires the definition of the parameter k, which directly influences the classification performance. To do this, an internal validation by means of 10-fold cross validation was performed on training data, and the k was selected by comparing the classification accuracy achieved for different values [49]. For WT1, a quantification model to estimate odor concentration at the fenceline was also built, considering the base10 logarithm of concentration values. The choice of using log transformation of concentration values was intended to aid a linear relationship between the independent and dependent variables before fitting linear regression models for odor quantification. Moreover, since log transformation allows compressing the differences between larger and smaller values, it resulted effective in reducing the range of odor concentration values obtained for training samples, improving the stability of linear models. As a drawback, log transformation may not always be helpful in dealing with outliers present in the data set, as it only works on extreme values that are within a certain range of the rest of the data points. Thus, verifying the robustness of the regression models by means a residuals analysis is fundamental because residuals (i.e., differences between observed and predicted values) are representatives of the goodness of the models, pointing out bias associated with the presence of outliers and/or valueless predictors. Residuals need to be completely independent each other's, normally and linearly distributed, not specifically correlated with some of the independent variables and their variances need to be sparce distributed among the predicted values in order to avoid any type of systematic errors included in the regression models negatively affecting the performance of the model.

In this study, the influence of different odor fingerprints of samples representatives of various plant odor sources on the precision of the quantification model was investigated. For this purpose, the novel double phase model, consisting of a classifier and three PLS regressions, i.e., one for each landfill odor class (model A), and a simpler regression model, as the ones typically described in the literature (model B), were compared.

PLS models were implemented based on responses of MOS, H_2_S and NH_3_ sensors relevant to the analysis of training samples at increasing concentrations and optimized by 10-fold Cross Validation (CV) selecting the configuration allowing obtaining the lower root mean squared error for prediction (RMSEP) [51]. Concerning model A, regression models for Leachate, Landfill Gas and Fresh Waste classes involved 6-, 5- and 3-PLS components, respectively. This approach allowed accounting, in the tuning of quantification models, for the different odor fingerprints associated to training samples having the same odor concentrations but belonging to different landfill odor sources characterized by different chemical compositions.

For model B, training data obtained from e-nose sensors (i.e., MOS, H_2_S and NH_3_) were processed as a whole dataset at increasing odor concentrations, not considering their odor class. In this case, based on the RMSEP evaluation achieved on 10-fold CV, a 4-PLS components model was selected.

### 2.4 Field testing

During monitoring, performance tests were carried out in the field to verify e-noses capability to detect, classify and quantify odors from the landfill. This phase was fundamental to validate the models implemented on training data, characterized by limited sample size, which are prone to risk of overfitting.

New samples were collected at emissions, analyzed by olfactometry, and diluted with odorless ambient air to obtain samples at different concentration levels within the TS concentration range. Table 1 lists, for each class, the odor concentration of samples used. As for training, the concentration levels differ for the two e-noses, since they are expected to be exposed to different odor concentrations during monitoring: the WT1 will presumably analyze more odorous air compared to the EOS 507F. Test samples were presented to e-noses by alternating diluted odor samples at different concentrations to odorless ambient air.

**Table 1:** Odor concentrations of the odor samples used for field testing.

Odor class	Tested odor concentration levels [ou_E_/m^3^]	
	WT1	EOS 507F
Landfill gas	130 - 200–317 - 500 - 610	25 - 33–49 - 57–80 -165
Fresh waste	60 - 150–260 - 280	27 - 35–67 - 164
Leachate	170 - 250 - 450	25 - 30–60 - 80 - 175

The detection and classification performances were expressed as accuracy indexes (AI_detection_ and AI_classification_), defined as the ratio between the number of correctly classified measures and the total number of measures [38]. Moreover, the IOMS LDL and Lower Classification Limit (LCL), expressed in ou_E_/m^3^, representing the lowest odor concentration at which the IOMS is capable of correctly detecting and classifying landfill odors respectively, were evaluated [38].

Performance testing also involved the verification of the WT1 capability to provide a reliable estimation of odor concentration at landfill fenceline by comparing odor concentrations estimated by the IOMS with the reference concentration assessed by dynamic olfactometry. The high uncertainty associated with the reference method, which is estimated to be around a factor of 3 [47], implies the need to develop specific procedures to evaluate the quality of the odor estimations by e-noses. Different approaches for model comparison based on regression and statistical evaluation have been proposed in the literature [52,53]. Among them, Bland-Altman (B&A) model, widely employed for clinical applications and recently applied also in environmental studies [32,35,54], can be considered suitable for this purpose. B&A compares two quantitative measurements relying on the quantification of their agreement, which is expressed in terms of mean difference (Bias) and Limits of Agreement (LoA) [55]. In this study, we applied B&A to assess and compare the performances of models A and B in quantifying odors with the dynamic olfactometry.

### 2.5 Ambient air monitoring

E-noses were installed for about 1 month at the monitoring sites and continuously analyzed ambient air. The EOS 507F analysis cycle includes an interruption of the measurements every 30 h for automatic calibration with the internal reference and baseline restoration. All the data recorded during monitoring were processed by models built on the training data to provide a qualitative outcome at both monitoring site and an estimation of the odor concentration at fenceline.

Based on IOMS predictions at receptors, the landfill odor impact, which is expressed as the frequency with which odors attributable to the landfill are detected, was assessed [38]. In this regard, the EOS 507F detections were evaluated in combination with meteorological conditions (i.e., wind speed and direction): detections of landfill odors occurring when the wind had an incompatible direction with the location of the receptor were considered as false positives and excluded from the calculation of the landfill odor impact. The EOS 507F detections were further compared with the WT1 responses to verify when the EOS 507F odor detections at the receptor corresponded to odor detections of the WT1 at the plant fenceline [37].

The acceptability of the odor impact assessed by the IOMS at the receptor can be evaluated referring to the Technical Instructions on Air Quality Control (TA Luft) on odor inputs [56], which fixes a limit of acceptable *odor hours* at 10 % for residential or mixed areas, and at 15 % for industrial or agricultural areas. Even though this regulation refers to a different technique, i.e. to field inspection (EN16841:2016-Part 1 [57]), it is often used as a reference also for odor impact assessments carried out with IOMS, since to date there are no other more specific guidelines [38,42].

## 3 Results & discussion

### 3.1 Training set

#### 3.1.1 Definition of Lower Detection Limit

Table 2 summarizes the LDL values determined for the two e-noses for each landfill odor class as well as concentration ranges considered. WT1 LDL turned out to be about 60 ou_E_/m^3^ for Landfill Gas and about 50 ou_E_/m^3^ for Fresh Waste and Leachate. The LDL of EOS 507F, which is specifically designed for operating at receptors and thus to recognize diluted odors, turned effectively out to be lower: it proved to be able to detect landfill odors down to ca. 30 ou_E_/m^3^ for Fresh Waste and Leachate and ca. 20 ou_E_/m^3^ for Landfill Gas.

**Table 2:** Summary of Lower Detection Limit (LDL), odor concentration range and number of samples considered for training the two instruments towards the WTP odor classes.

Odor class	WT1			EOS 507F		
	LDL [ou_E_/m^3^]	Odor concentration range [ou_E_/m^3^]	N. training samples	LDL [ou_E_/m^3^]	Odor concentration range [ou_E_/m^3^]	N. training samples
Fresh Waste	50	50–310	8	*30*	30–260	8
Landfill gas	60	60–700	10	20	20–300	10
Leachate	50	50–620	10	30	30–230	10

#### 3.1.2 Exploratory analysis

**Fig. 3:**
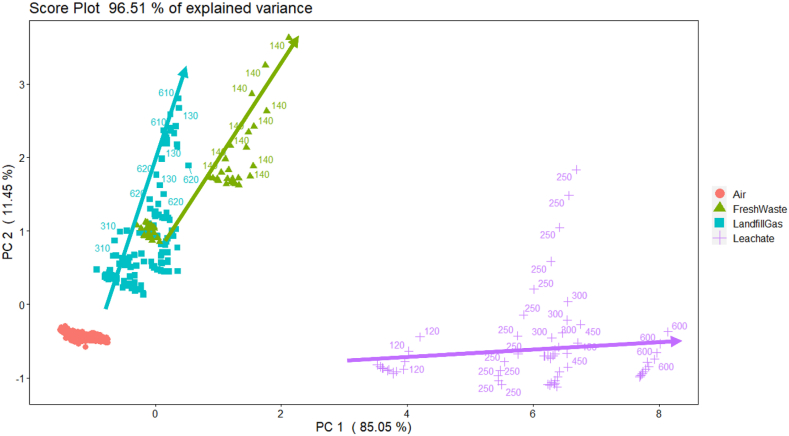
Principal Component Analysis (PCA) score plot relevant to WT1 Training Set. The points labels indicate the odor concentration of samples, arrows indicate the direction along which the odor concentration increases.

Fig. 3 reports the PCA score plot relevant to the WT1 TS. Landfill samples are well distinguished from odorless ambient air and cluster in different areas of the plot. Arrows in Fig. 3 indicate the direction along which sample odor concentration increases for each odor class. The fact that points having similar odor concentrations but belonging to different classes cluster in different regions, on one hand proves the good e-nose capability to discriminate landfill odors, but it also suggests the need of developing specific quantification models for each odor class.

### 3.2 Field performance testing

#### 3.2.1 Fenceline

15 odorless air samples and 12 landfill samples independent from TS (i.e., 3 samples belonging to Leachate, 5 samples to Landfill Gas and 4 samples to Fresh Waste) were presented to IOMS for performance validation, according to the odor concentration values expected at the plant fenceline (Table 1, Table 3). Moreover, other 5 landfill samples were prepared at lower concentrations (i.e., 50 ou_E_/m^3^ for Fresh Waste, 40 and 50 ou_E_/m^3^ for Landfill Gas and 40 and 50 ou_E_/m^3^ for Leachate) and analyzed by the IOMS to investigate the LDL and LCL for each class of odor. Results obtained confirmed the LDL assessed in the training phase (i.e., 50 ou_E_/m^3^), which coincides with the LCL for all landfill classes: the WT1 proved to be capable to detect and correctly classify samples having an odor concentration ≥50 ou_E_/m^3^ (Table 3).

**Table 3:** Summary of field performance testing results: Number of field testing samples considered, Lower Detection Limit (LDL), Lower Classification Limit (LCL) and Accuracy Indexes of the two instruments.

Odor class	WT1			EOS 507F		
	N. field testing samples	LDL (ou_E_/m^3^)	LCL (ou_E_/m^3^)	N. field testing samples	LDL (ou_E_/m^3^)	LCL (ou_E_/m^3^)
Landfill gas	5	50	50	6	20	30
Fresh waste	4	60	60	4	30	30
Leachate	3	50	50	5	30	30

**E-Nose Task**	**Accuracy Index**	**95 % CI**	**Accuracy Index**	**95 % CI**
Detection	94 %	88 %–97 %	95 %	84.5 %–99.4 %
Classification	93 %	88 %–96 %	91 %	73 %–98.9 %

More in detail, the WT1 proved a very good capability to detect landfill odors with an AI_detection_ of 94 %(CI_95 %_ 88%–97 %), and to discriminate the different classes with an AI_classification_ of 93 %(CI_95 %_ 88%–96 %) (Table 3).

**Fig. 4:**
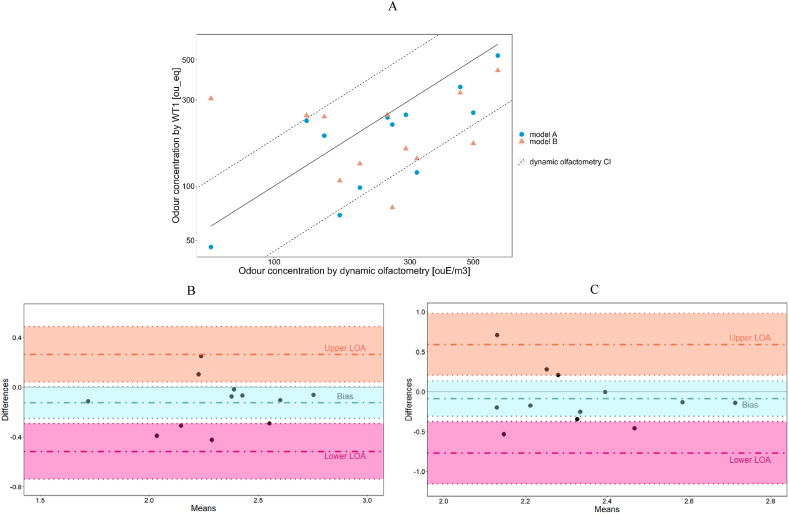
Evaluation of the WT1 quantification performance: Comparison of model A and model B predictions with dynamic olfactometry (A); Bland-Altman plots for model A (B) and model B (C).

Furthermore, field tests at fenceline focused on the verification of the odor quantification performance by comparing the WT1 estimations of the odor concentrations of the field test samples, based on model A or B, with the results of dynamic olfactometry (Table 4A). Fig. 4A graphically compares in logarithmic scale odor concentrations estimated by e-nose models versus measured concentrations by dynamic olfactometry: the diagonal in the plot represents the strict correspondence between predicted and measured odor concentration values, while the dotted lines represent the confidence interval of the dynamic olfactometry. Concentrations estimated by model A better agrees with the reference odor concentration than those obtained by model B. Indeed, all field measurements fall within or very close to olfactometry CI_95 %_ limits. Conversely, some of the estimations provided by model B exceed such limits, as can be noticed looking at the concentration values estimated for two Fresh Waste samples highlighted in red in Table 4A.

**Table 4:** Evaluation of the quantification performance: Comparison between the odor concentration assessed by dynamic olfactometry and e-nose estimations. Predictions falling within the confidence interval of the dynamic olfactometry are highlighted in green, while the ones falling outside the confidence interval of the dynamic olfactometry in red (A); Predictive performance of model “A” and model “B” computed by means of Bland-Altman model (B).

Sample	Odor class	Dynamic olfactometry		WT1 analysis		
		Odor concentration (ou_E_/m^3^)	Confidence interval 95 % (ou_E_/m^3^)	Classification	Concentration (Model "A") (ou__eq_)	Concentration (Model "B") (ou__eq_)
1	Leachate	170	75–330	Leachate	69	107
2	Leachate	250	110–480	Leachate	240	247
3	Leachate	450	200–870	Leachate	354	328
4	Landfill Gas	317	140–610	Fresh Waste	119	142
5	Landfill Gas	610	270–1200	Landfill Gas	528	437
6	Landfill Gas	200	88–390	Landfill Gas	98	133
7	Landfill Gas	500	220–970	Landfill Gas	255	172
8	Landfill Gas	130	57–250	Landfill Gas	230	246
9	Fresh Waste	150	66–290	Fresh Waste	190	242
10	Fresh Waste	260	110–500	Fresh Waste	219	76
11	Fresh Waste	60	26–120	Fresh Waste	46	305
12	Fresh Waste	290	130–560	Fresh Waste	248	161

B&A parameters	Model “A” vs Dynamic Olfactometry		Model “B” vs Dynamic Olfactometry	
	Logarithmic Differences	Multiplicative factors	Logarithmic Differences	Multiplicative factors
Bias	−0.13		−0.09	
Upper LoA CI 95 %	0.26	1.8x	0.59	3.9x
Lower LoA CI 95 %	−0.52	0.3x	−0.77	0.17x

As further evaluation, B&A method was applied to include the high uncertainty related to olfactometry in the comparison of two quantification models [55]. Since B&A assumes a normal distribution, the log-normal distribution of the differences between the models was tested by Shapiro-Wilk test to check if the data fulfil the hypothesis. Indeed, B&A method requires the normal distribution of the differences calculated between the dynamic olfactometry concentration values and the corresponding odor concentration values predicted by the quantification models (i.e., Model A and Model B), because such differences represent the input values of the model to calculate Bias and Limits of Agreement (LoA). In both cases (i.e., model A vs. olfactometry and model B vs. olfactometry), a p-value significantly higher than the significance threshold of 0.05 (i.e., ∼0.9) was obtained, meaning that the hypothesis of normal distribution of the differences cannot be rejected, and thus justifying the application of the B&A method to the dataset [58]. Fig. 4B and C show the results of B&A obtained for models A and B, respectively. They illustrate Bias (blue dashed line), Upper LoA (orange dashed line) and Lower LoA (pink dashed line) with relative CI_95 %_ (corresponding-coloured boxes) calculated based on standard deviation, while black dots represent field measurements. Bias is close to 0.1 for both models: e-nose predictions are like odor concentrations assessed by olfactometry. However, the visual inspection of B&A plots points out that models A and B cannot be considered comparable because of significant differences in the LoA. Indeed, black dots in Fig. 4C are characterized by greater deviations from the Bias and mainly close to Lower or Upper LoA.

Aiming to simplify the comparison of results achieved by models A and B with odor concentration by reference method, B&A LoA were also expressed in terms of multiplicative factors (Table 4B). Such multiplicative factors directly express the deviation of predicted values [ou_eq] from reference odor concentration [ou_E_/m^3^] and can be directly compared with the acceptability criteria defined by EN 13725:2022 for two consecutive measurements (i.e., intermediate precision <3) [35].

For model A, Lower and Upper LoA are 0.3x and 1.8x, respectively, indicating that the 95 % of the predictions obtained by the model are within a factor 3 from the reference value obtained by olfactometry. For model B, LoA are equal to 0.17x and 3.9x respectively, resulting in a multiplicative factor of 4. These results clearly highlight different levels of agreement of the models A and B with dynamic olfactometry. More in detail, concentrations estimated by model A turned out to be closer to the odor concentration assessed by the reference method. Therefore, in case of odor sources characterized by very different chemical compositions, odor classification prior to quantification is suggested to considerably improve the accuracy and the reliability of the odor concentration predictions.

#### 3.2.2 Receptor

The IOMS installed at receptor was also tested in the field: 13 odorless air samples and 15 landfill samples, whose odor concentrations are listed in Table 1, were analyzed. As for WT1, field tests confirmed the LDL towards landfill odors assessed during the training: about 20 ou_E_/m^3^ for Landfill Gas samples, and 30 ou_E_/m^3^ for Fresh Waste and Leachate samples. Field tests proved the EOS 507F capability to correctly classify Leachate and Fresh Waste samples having an odor concentration very close to the LDL, thereby for those classes the LCL coincides with the LDL. On the contrary, the LCL towards Landfill Gas is slightly higher than the LDL, i.e. ca. 30ou_E_/m^3^. The knowledge of the LCL is crucial especially for monitoring at receptors, for which IOMS detections should ideally be comparable with observations from citizens.

EOS 507F testing resulted in an AI_detection_ and AI_classification_ of 95 % (CI_95 %_ 84.5%–99.4 %) and 91 % (CI_95 %_ 73%–98.9 %) respectively (Table 3), thus proving very good detection and classification performances.

### 3.3 Monitoring results

This study focuses on the description of the approach adopted to develop odor quantification models. However, in this section we decided to report the results of the odor monitoring activities to give an idea of the e-nose outputs, and what they are used for.

#### 3.3.1 Fenceline

**Fig. 5:**
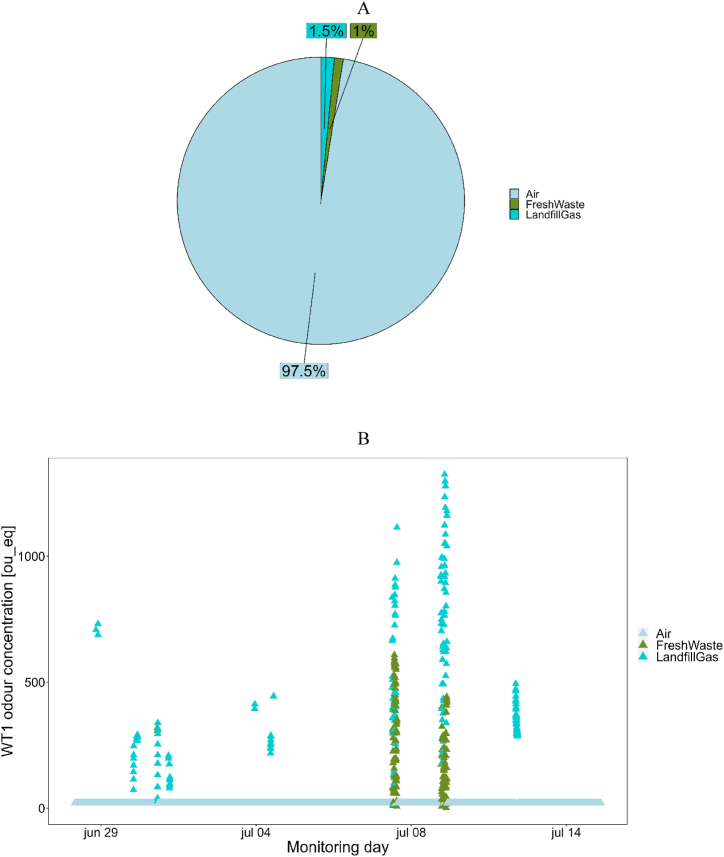

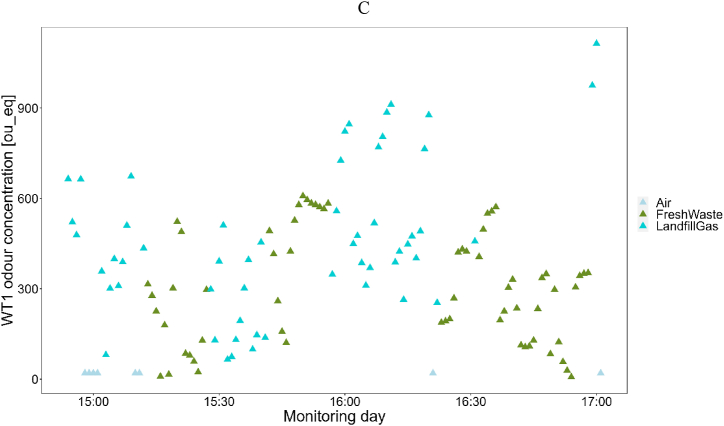
Results of monitoring at fenceline: Frequency of detection of odors from landfill at the fenceline (A); Odor concentration values estimated overall the monitoring period (B); Zoom of the odor concentration values estimated on July 8th (C).

At fenceline, the presence of landfill odors was detected for about 2.5 % of the monitoring period: the WT1 detected odors attributable to Fresh Waste and Landfill Gas classes for respectively 1 % and 1.5 % of the monitoring, while it detected Air for about 97.5 % of the monitoring (Fig. 5A). As expected, no detections related to Leachate occurred because of the considerable distance of the leachate tanks from the monitoring site and their relatively low odor concentration compared to the other landfill emission sources.

After classification, the odor concentration at fenceline was estimated by the quantification model A (Fig. 5B). In general, the odor concentrations estimated turned out to be lower than 500 ou__eq_ when Fresh Waste odor was recognized, while odors events attributed to Landfill Gas reached concentrations from 50 ou__eq_ up to 1300 ou__eq_. These high concentration levels could be related both to the proximity of the e-nose installation site to a landfill gas collection station, but also to the fact that landfill gas is usually characterized by higher odor concentrations than fresh waste. Fig. 5C shows in detail the detections recorded on July 8th to aid the visualization of the occurrence of such frequent odor events.

#### 3.3.2 Receptor

**Fig. 6:**
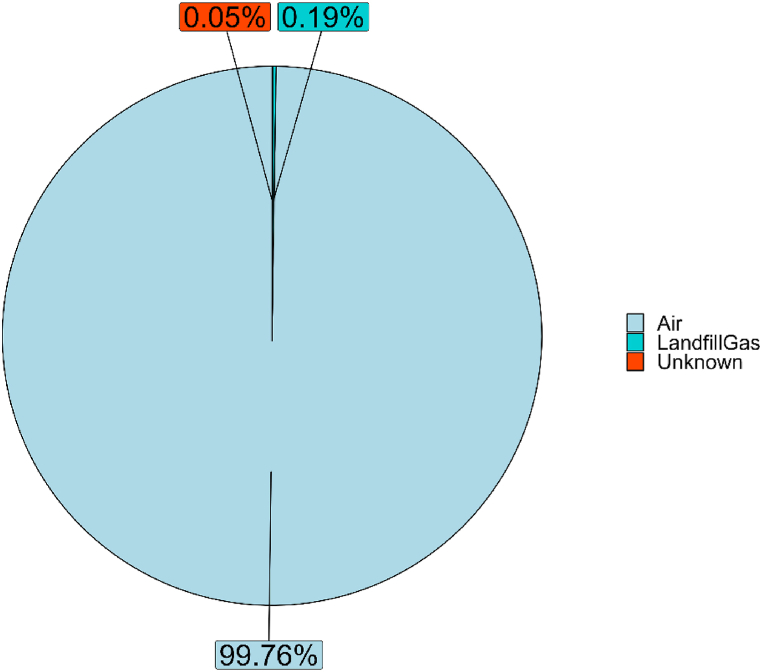
Frequency of detection of odors from landfill at receptor.

EOS 507F detections during the monitoring were evaluated in combination with weather conditions and WT1 detections at the fenceline, to identify possible false positives to be excluded from the assessment of landfill odor impact [39]. Then, e-nose detections purged from false positives were expressed in terms of detection frequency. The EOS 507F detected the presence of odors for 0.24 % of the monitoring period, with 0.19 % being odors attributable to Landfill Gas. The other 0.05 % of the odor detections were classified as *unknown*, since they could not be attributed to any of the landfill odor classes considered during the training (Fig. 6).

### 3.4 Comparison between receptor and fenceline odor events

To evaluate the possibility to identify specific thresholds for the odor concentrations at fenceline that might result in odor events at the receptor, landfill odor events at receptor during the monitoring were analyzed in combination with information (i.e., classification and odor concentration) provided by the WT1 and meteorological conditions. The comparison highlighted the existence of a correlation among odor presence at fenceline and the occurrence of odor episodes attributable to the landfill at the receptor detected by EOS 507F. Table 5 lists the odor events relevant to the monitoring: for each odor event, day, duration, odor type and concentration value estimated at fenceline, and meteorological conditions are reported.

**Table 5:** Comparison between landfill odor events at the receptor and WT1 detections at the fenceline with meteorological conditions (wind speed: “absent” $v_{wind}\leq 1m/s$, “weak” $1\;m/s<v_{wind}\leq 2m/s$, “strong” $2\;m/s<v_{wind}\leq 5m/s$).

N°	Date	Duration [min]	Class detected at receptor	Class detected at fenceline	Concentration at fenceline [ou_eq]	Wind speed	Wind direction
1	29/06/2020	60	Air	Landfill gas	710	Weak	From West-NorthWest (WNW) to East-SouthEast (ESE)
2	01/07/2020	10	Air	Landfill gas	150	Weak	From North (N) to South (S)
3	01/07/2020	20	Air	Landfill gas	270	Weak	From North (N) to South (S)
4	02/07/2020	10	Air	Fresh Waste	50	Weak	From NorthWest (NW) to SouthEast (SE)
5	02/07/2020	20	Air	Landfill gas	190	Weak	From SouthWest (SW) to NorthEast (NE)
6	02/07/2020	20	Air	Landfill gas	90	Weak	From North (N) to South (S)
7	03/07/2020	60	Unknown	Air	20	Weak	From North (N) to South (S)
8	04/07/2020	10	Air	Landfill gas	400	Weak	From SouthEast (SE) to NorthWest (NW)
9	06/07/2020	90	Landfill gas	Landfill gas	300	Weak	From North (N) to South (S)
10	08/07/2020	40	Air	Landfill gas	320	Weak	From South (S) to North (N)
11	08/07/2020	25	Air	Fresh Waste	120	Weak	From South (S) to North (N)
12	08/07/2020	25	Air	Landfill gas	520	Weak	From South-SouthEast (SSE) to North-NorthWest (NNW)
13	08/07/2020	40	Air	Fresh Waste	210	Weak	From South (S) to North (N)
14	09/07/2020	60	Landfill gas	Landfill gas	700	Weak	From North (N) to South (S)
15	11/07/2020	40	Air	Landfill gas	340	Strong	From NorthEast (NE) to SouthWest (SW)

During the monitoring, 14 odor events, attributed to Landfill Gas and Fresh Waste classes, were detected at plant fenceline. Only in two cases, landfill odors were also perceived at receptor. In both cases, the WT1 estimated concentrations above 300 ou_eq and classified the odor as Landfill Gas, which corresponded also to the odor class recognized at receptor. However, it is not possible to state that every time the odor concentration measured at fenceline exceeds 300 ou__eq_ an odor event is registered at receptor. Indeed, in case of unfavorable wind conditions (e.g., wind blowing in an incompatible direction with respect to the receptor position – red highlighted rows in Table 5), no odor event was recorded at the receptor even if more than 300 ou__eq_ were measured at the fenceline (blue highlighted rows in Table 5).

Moreover, there are some cases in which, despite a high odor concentration (i.e. > 300 ou__eq_) is measured at fenceline and the wind direction is compatible with the perceptions of odors from the landfill at the receptor, no odor is detected by the EOS 507F. This is for instance the case of event no. 15, characterized by strong wind (Table 5): with high wind speed, the atmospheric dilution of pollutants is usually improved, giving that odors are presumably diluted below the detection threshold and are thus not perceived at receptor. These observations suggest that setting a “fixed” alarm threshold at 300 ou__eq_ at fenceline would not be an effective solution to predict odor events at receptor, since the exceedance of such threshold would not necessarily result in an odor event in the surroundings of the plant, for instance because of unfavorable meteorological conditions for the detection of odors from the landfill at receptors (e.g., wind blowing in the opposite direction). The definition of “variable” critical thresholds depending on wind speed and direction may result in a more effective approach in such conditions. Therefore, further developments of the research should focus on the development of a specific system able to combine in real-time the information about the odor concentration with the meteorological conditions and the atmospheric dispersion capability, to effectively evaluate the probability that odors are perceived at a certain distance from the plant [59].

## 4 Conclusions

This paper proposes a novel approach to train and validate an e-nose for the real-time measurement of odor concentration. The novelty of the approach is related to the development of a double step model, which first operates a qualitative characterization of the ambient air and then estimates the odor concentration based on specific regression models for each odor class.

Even though based on limited dataset, preliminary results obtained prove that the introduction of a classification step prior to quantification significantly improves the accuracy of the estimations, especially when the odor sources are characterized by very different chemical compositions, leading to predicted odor concentration values falling within the confidence interval of the reference measurement method, i.e., dynamic olfactometry (EN 13725:2022). Statistical evaluations pointed out the reliability of the results obtained, but still further studies should focus on further validating evidence here reported considering extended datasets.

Moreover, this study further proposes a method based on B&A approach to evaluate the goodness of odor concentration estimations by e-nose compared with values assessed by reference method (i.e., dynamic olfactometry) able to include in the evaluations also the uncertainty associated to the dynamic olfactometry. LoA obtained for double step quantification model are within a factor of 3 from the true value of concentration assessed by dynamic olfactometry, which can be considered acceptable based on EN 13725:2022.

This study also involved the use of a second e-nose installed 2 km South from the landfill along prevalent wind direction, aiming to make some preliminary considerations about the existence of a correlation between concentrations measured at fenceline and the detections of landfill odors at the receptor. The comparison of e-noses outcomes during the monitoring suggests the need to consider the meteorological conditions to define suitable variable alarm threshold to be set at plant fenceline effectively correlated to the probability of occurrence of situations that may lead to the perception of odors outside of the plant in case of exceedance. Future studies should deeply investigate this aspect to define rigorous approaches for alarm threshold definition.

## Data availability statement

The data that has been used is confidential.

## CRediT authorship contribution statement

**Beatrice Julia Lotesoriere:** Data curation, Writing – original draft, Writing – review & editing, Validation, Visualization. **Carmen Bax:** Methodology, Project administration, Supervision, Writing – review & editing, Conceptualization. **Laura Capelli:** Project administration, Supervision, Writing – review & editing.

## Declaration of competing interest

The authors declare that they have no known competing financial interests or personal relationships that could have appeared to influence the work reported in this paper.
